# Ingested Engineered Nanomaterials Affect the Expression of Mucin Genes—An In Vitro-In Vivo Comparison

**DOI:** 10.3390/nano11102621

**Published:** 2021-10-06

**Authors:** Gerrit Bredeck, Angela A. M. Kämpfer, Adriana Sofranko, Tina Wahle, Veronika Büttner, Catrin Albrecht, Roel P. F. Schins

**Affiliations:** Particle Research, IUF—Leibniz Research Institute for Environmental Medicine, 40225 Düsseldorf, Germany; gerrit.bredeck@iuf-duesseldorf.de (G.B.); angela.kaempfer@iuf-duesseldorf.de (A.A.M.K.); adriana.sofranko@iuf-duesseldorf.de (A.S.); tina.wahle@iuf-duesseldorf.de (T.W.); veronika.buettner@rwth-aachen.de (V.B.); catrin.albrecht@sachsen-anhalt.de (C.A.)

**Keywords:** nanoparticles, mucus barrier, inflammation, gut, intestine, safety evaluation, hazard assessment

## Abstract

The increasing use of engineered nanomaterials (ENM) in food has fueled the development of intestinal in vitro models for toxicity testing. However, ENM effects on intestinal mucus have barely been addressed, although its crucial role for intestinal health is evident. We investigated the effects of ENM on mucin expression and aimed to evaluate the suitability of four in vitro models of increasing complexity compared to a mouse model exposed through feed pellets. We assessed the gene expression of the mucins MUC1, MUC2, MUC5AC, MUC13 and MUC20 and the chemokine interleukin-8 in pre-confluent and confluent HT29-MTX-E12 cells, in stable and inflamed triple cultures of Caco-2, HT29-MTX-E12 and THP-1 cells, and in the ileum of mice following exposure to TiO_2_, Ag, CeO_2_ or SiO_2_. All ENM had shared and specific effects. CeO_2_ downregulated MUC1 in confluent E12 cells and in mice. Ag induced downregulation of Muc2 in mice. Overall, the in vivo data were consistent with the findings in the stable triple cultures and the confluent HT29-MTX-E12 cells but not in pre-confluent cells, indicating the higher relevance of advanced models for hazard assessment. The effects on MUC1 and MUC2 suggest that specific ENM may lead to an elevated susceptibility towards intestinal infections and inflammations.

## 1. Introduction

Within the last decades, the utilization of engineered nanomaterials (ENM) in food products and food packaging has increased dramatically [1]. As a food additive, TiO_2_ (European food additive code E171), which typically contains up to 50% nanoparticles, is used as a whitener and UV absorbent [2,3,4], whereas Ag (E174) is particularly used for its antimicrobial properties [2,5,6,7], and amorphous SiO_2_ (E551) is utilized as an anti-caking agent and to stabilize oil-water emulsions [2,8]. In addition to the intentional supplementation, ENM can enter the food chain as contaminants, as has been shown for CeO_2_ [9,10,11,12].

Hence, humans are increasingly orally exposed to ENM, which has raised safety concerns [13,14]. In the context of ENM safety evaluation, many authors highlighted the importance to account for the intestinal mucus barrier [15,16,17,18]. On the one hand, the interaction of mucus with ENM, such as the entrapping and the formation of a corona, have been extensively researched both in vitro and in vivo [19,20,21,22]. On the other hand, however, effects on the mucus barrier itself, especially on the expression of mucin genes, have received surprisingly little attention. So far, only a few studies have pointed out altered expression levels and differences in mucus compositions following ENM exposure [23,24,25,26,27]. Potential deleterious effects of ingested ENM on the expression and secretion of mucins are of high concern since the functionality of the mucus barrier is crucial for intestinal health. An impairment of the mucus barrier’s capacity to segregate the luminal content from the intestinal epithelium can lead to the continuous induction of inflammatory processes and has been demonstrated in subjects with inflammatory bowel diseases [28,29,30]. 

Two different types of mucins build the mucus barrier: secreted mucins derived from goblet cells and transmembrane mucins that are also produced by enterocytes [31,32,33]. In the healthy intestine, the secreted mucin (MUC)2 and the transmembrane mucins MUC3, MUC12, MUC13 and MUC17 are the most prominent [34]. In the course of intestinal inflammatory diseases, particularly the predominant gastric mucins MUC1 and MUC5AC, but also other mucins such as MUC20 were described to be differentially expressed [34,35,36]. The importance of the constitutive expression of mucins has been highlighted by mouse models deficient of MUC1, MUC2, and MUC13, revealing elevated susceptibilities towards intestinal infections, inflammations and spontaneous tumors [37,38,39,40,41,42].

The safety concerns of ENM, in combination with the endeavor to replace animal testing, strongly accelerated the development of advanced in vitro models to investigate intestinal toxicity [43]. Most-commonly, the cell lines Caco-2 and HT29-MTX are used. When grown at confluence, they differentiate into physiologically relevant enterocyte- and goblet-like cell types, respectively [44,45,46]. In a next step, researchers combined both cell lines in order to obtain an enterocyte model covered with a mucus layer [17,43,47,48,49]. Moreover, other cell lines such as differentiated THP-1 cells and RajiB cells have been incorporated to account for macrophages or to induce a microfold (M)-cell like transformation in Caco-2 cells, respectively [17]. To our knowledge, an in vitro to in vivo comparison of ENM effects using the same ENM in in vitro and in vivo models, as presented in this study, has yet to be performed. 

The first aim of this study was to assess whether four well-investigated model ENM have specific, potentially hazardous effects on the intestinal expression of the mucins MUC1, MUC2, MUC5AC, MUC13 and MUC20. In view of the importance of inflammation in gut homeostasis and the well-described inflammatory effects of specific ENM we concurrently evaluated the expression of the proinflammatory cytokine interleukin (IL)-8 and its murine equivalent homologs macrophage inflammatory protein (Mip)-2 and keratinocyte-derived chemokine (Kc). We observed trends of effects that all investigated ENM had in common as well as ENM specific effects on the mucus profile that may pose a hazard to human health. A complementary goal of our study was to ascertain whether in vitro models of increased complexity can mirror the in vivo effects of ENM on the expression of the abovementioned mucins and whether the induction of an inflamed-like condition may influence ENM effects in vitro. Growth at confluence particularly improved the suitability of in vitro models. The inflamed-like state came along with a changed mucin profile but not with an increased susceptibility towards the ENM. 

## 2. Materials and Methods

### 2.1. Murine Ileal Samples

Murine ileal samples were obtained from two independent feeding studies with C57BL/6J mice, as we previously described in detail [50]. These studies were performed with the same batches of the ENM (i.e., TiO_2_ P25, Ag-PVP, CeO_2_, and SiO_2_) as those that were used for the in vitro tests. The studies were approved by the Landesamt für Natur, Umwelt und Verbraucherschutz (LANUV, NRW, Germany) with the reference numbers 84-02.04.2017.A338 and 84-02.04.2013.A443. Briefly, in one study female and male animals were fed with feed pellets containing TiO_2_ (1% *w/w*) and Ag (0.2% *w/w*) ad libitum for 28 days. In the other study, female animals were fed with feed pellets containing CeO_2_ and SiO_2_ (both at 1% *w/w*) ad libitum for 21 days. The animals were sacrificed on the last day of exposure and ileal tissues were obtained by dissection and subsequently snap frozen in liquid nitrogen and stored at −80 °C until analysis.

### 2.2. Chemicals and Reagents

Minimum Essential Medium (MEM), Dulbecco’s Modified Eagle Medium (DMEM), RPMI-1640 medium, 2-mercaptoethanol (ME), fetal calf serum (FCS) for THP-1 cells, sodium pyruvate, phosphate buffered saline (PBS) and Prolong Gold Antifade Reagent were purchased from Thermo Fisher Scientific. FCS for Caco-2 and HT29-MTX-E12 cells, Penicillin/Streptomycin (P/S), L-glutamine, non-essential amino acids (NEAA), D-glucose, trypsin, phorbol 12-myristate 13-acetate (PMA), interferon gamma (IFN-γ), lipopolysaccharides (LPS), accutase, the Cell Proliferation Reagent WST-1, acetic acid, Alcian blue (1% in 3% acetic acid), periodic acid, Schiff’s reagent, sodium meta bisulfite, the Roche High Pure RNA Tissue Kit and the amplification grade DNase I Kit were purchased from Sigma-Aldrich/Merck. Ethanol was purchased from Roth. Nuclease free water was purchased from Qiagen. The iScript^TM^ cDNA Synthesis Kit and the iQ^TM^ SYBR^®^ Green Supermix were purchased from Bio-Rad. Primers for qPCR were purchased from Eurofins.

### 2.3. Engineered Nanomaterials

The same batches of TiO_2_ P25, polyvinylpyrrolidone-coated Ag (Ag-PVP), CeO_2_ and fumed SiO_2_ ENM as described previously [50] were used and the formerly reported characteristics are presented in Table S1. For the use in experiments, ENM were suspended in sterile, deionized water (dH_2_O) to a concentration of 3.57 or 4.00 mg/mL. The suspensions as well as dH_2_O not containing ENM were sonicated for 10 min using a Branson Sonifier 450 at a duty cycle of 0.2 s and an output of 240 W. 

### 2.4. Sedimentation of Engineered Nanomaterials

The delivered ENM doses in E12 cell monoculture experiments, performed in 6-well plates, were estimated using the In Vitro Diffusion and Dosimetry model (ISDD) [51]. The input parameters described in Kämpfer et al. [52] were applied and, where necessary, adapted to the 6-well format and complemented for CeO_2_ and SiO_2_. The effective agglomerate diameters of CeO_2_ and SiO_2_ were measured as described in Kämpfer et al. [52]. Briefly, ENM were suspended in exposure medium to 150 µg/mL and the Z-average and polydispersity index (PDI) were measured using a Zetasizer NanoZS (Malvern Pananalytics, Malvern, UK). 

### 2.5. Cell Culture

Caco-2 (DSMZ, ACC169) cells were cultured in MEM (containing NEAA) substituted with 20% FCS, 1% P/S and 1% L-glutamine. HT29-MTX-E12 (ECACC through Sigma, 12040401) cells (hereinafter ‘E12 cells’), were maintained in DMEM (containing L-glutamine) substituted with 10% FCS, 1% P/S and 1% NEAA. Cells of both cell lines were regularly subcultured upon reaching about 80% confluence every three to four days. For experiments, Caco-2 and E12 cells were used between passages 3 and 30. THP-1 (ATCC, TIB-202) cells were cultured in RPMI 1640 medium (containing L-glutamine and 25 mM HEPES) substituted with 10% FCS, 1% P/S, 1 mM sodium pyruvate, 0.7% D-glucose and 50 nM ME. THP-1 cells were maintained at densities between 2*10^5^ and 8*10^5^ cells/mL. For experiments, THP-1 cells were used at passages 5–15 after thawing.

Experiments on E12 cell monocultures were performed two days, 11 days or 22 days post-seeding. The seeding density specified in Table 1 was used regardless of the culture period. After 4–5 days, 100% confluence was reached. Cells grown for two days will be termed ‘pre-confluent’. To obtain confluent cultures, E12 cells were maintained for 11 days or 22 days as indicated. The medium was changed three times per week. The medium was replaced with exposure medium, DMEM substituted with 1% FCS, 1% P/S and 1% NEAA, 16–20 h before exposure.

The exposure of E12 cells was performed in 6-well plates for mucus visualization and gene expression analysis and in 96-well plates for cytotoxicity assessment. The cell numbers and volumes of medium were adjusted to the size of the well surfaces so that equal exposure concentrations in μg/mL and μg/cm^2^ were obtained for both plate formats (Table 1).

The triple cultures in stable/healthy and inflamed state were prepared as described by Kämpfer et al. [52]. Briefly, 1.8*10^5^ Caco-2 and E12 cells per cm^2^ were seeded on 12-well transwell inserts (Falcon, 353103) at a ratio of 9:1 and cultivated for 21 days. On day 21, THP-1 cells were differentiated with 100 nM PMA for 24 h. The epithelial cells on the transwell inserts were basolaterally primed with 10 ng/mL IFN-γ for 24 h for the inflamed triple culture. On day 22, the differentiated THP-1 cells were detached, seeded into 12-well plates at a 1.8*10^5^ cells per well. At the same time, re-attached THP-1 cells were activated with 10 ng/mL LPS and IFN-γ for 4 h for the inflamed triple cultures. Subsequently, stable and inflamed triple cultures were started in parallel by transferring transwell inserts to the corresponding THP-1 containing wells. After incubation for 24 h, cells were exposed to particles for 24 h. 

### 2.6. Exposure Procedure

Particle suspensions were diluted in exposure medium to concentrations between 0 and 256 µg/mL, corresponding to doses between 0 and 80 µg/cm^2^. DMEM and MEM containing 1% FCS were used as exposure medium for E12 cell monocultures and the triple cultures, respectively. For negative controls and lower exposure concentrations, the added amount of sonicated dH_2_O was adjusted to correspond to the highest exposure concentration. E12 cell monocultures were exposure to the ENM suspensions for 4 h and 24 h, triple cultures were exposed for 24 h. 

### 2.7. WST-1 Cytotoxicity Assay

In E12 cell monocultures, the cell viability was assessed via WST-1 assay after 24 h exposure to 0, 10, 20, 40, and 80 µg ENM/cm^2^. The assay was performed as described by Kolling et al. [53] and based on the nanOxiMet project’s SOP “Cellular viability—WST-1 assay” (https://nanopartikel.info/data/projekte/nanOxiMet/SOP/nanOxiMet_SOP_WST-1-assay_V2.pdf, accessed on 23 August 2021). WST-1 reagent was added to the cells at a dilution of 1:5 in exposure medium and the absorbance at 450 nm and 630 nm was measured after incubation for 1 h. 

### 2.8. Alcian Blue Staining and Periodic Acid-Schiff Reaction

In order to visualize and localize the acidic and neutral mucus, produced by E12 cells, Alcian blue staining and periodic acid-Schiff (PAS) reaction were performed, respectively, as described in Kämpfer et al. [52]. Briefly, paraformaldehyde-fixed samples were pre-incubated with 3% acetic acid for 3 min and stained with 1% Alcian blue solution in 3% acetic acid for 30 min. Subsequently, washed samples were incubated with 1% periodic acid in dH_2_O for 10 min and with Schiff’s reagent for 15 min, protected from light. The samples were washed three times with sulphite water, once with dH_2_O, mounted with Prolong Gold Antifade reagent and analyzed by light microscopy. 

### 2.9. Gene Expression Analysis

The expression of MUC1, MUC2, MUC5AC, MUC13 and MUC20 in E12 monocultures and triple cultures, as well as of Muc1, Muc2, Muc5AC, Muc13 and Muc20 in murine ileal tissue was assessed. In addition, IL-8 and the murine homologs Mip-2 and Kc were assessed, respectively. To prepare the RNA isolation, E12 cell monocultures were washed with ice-cold PBS twice, detached with a cell scraper, collected, centrifuged at 4 °C and 300× *g* for 5 min and resuspended in PBS. To prepare the RNA isolation from triple cultures, cells were detached from filters with trypsin for 10 min, trypsin reaction was blocked with ice-cold MEM containing 20% FCS and cells were collected. The cells were washed by two repetitions of centrifuging at 4 °C and 300× *g* for 5 min, discarding the supernatant, and resuspending the cell pellet in PBS. Subsequently, RNA from E12 cells and from triple cultures was isolated using the Roche High Pure RNA Tissue Kit. Briefly, cells were lysed, RNA was bound to silica membrane spin columns, treated with DNase I, washed with high-salt concentration wash buffers containing ethanol, and eluted in PCR grade water. 

RNA from murine ileum was isolated as described in Kämpfer et al. [52]. Briefly, tissues were homogenized in Lysis Buffer using a Tissue Homogenizer II (Qiagen, Hilden, Germany) and ethanol was added to the lysates. The RNA was bound to silica membrane spin columns, treated with DNase I, washed with high-salt concentration wash buffers containing ethanol, and eluted in PCR grade water.

For in vitro and in vivo samples, RNA quantification, a second DNase I digestion step, reverse transcription, and quantitative PCR (qPCR) were performed as described in Kämpfer et al. [52]. Briefly, the RNA concentration was determined by measuring the optical density at 260 and 280 nm. The samples were treated with amplification grade DNase I. Two replicates of 0.5 µg RNA were reversely transcribed using the iScript^TM^ cDNA synthesis kit. A no reverse transcriptase control (nRTc) with one replicate of 0.5 µg RNA was performed in parallel to control for residual DNA. The duplicate cDNA samples were pooled. The cDNA and nRTc were diluted in nuclease free water by factor 15. When less RNA was available, the amount of RNA and the dilution factor were decreased proportionally. The iQ^TM^ SYBR^®^ Green Supermix was used for qPCR reactions. For in vitro samples, the human primers listed in Table S2 were used and β-Actin was analyzed as the reference gene. For in vivo samples, the murine primers listed in Table S3 were used and Rplp0 was analyzed as reference gene. qPCR reactions were performed and melt curves generated in triplicate for cDNAs and in one replicate for nRTcs using a MyiQ^TM^ Single-Color Real-Time PCR Detection System (Bio-Rad, Hercules, FL, USA). C_T_ values were determined using the Bio-Rad iQ5 software (v2.1). ENM exposure dependent changes in the gene expression were calculated using the ΔΔC_T_ method [54]. 

### 2.10. Statistical Analysis

ΔΔC_T_ values, fold changes, mean values and standard deviations (SD) or standard errors of the mean (SEM) were calculated with Microsoft Excel from the results of at least three independent experiments. For the gene expression analyses, ΔΔC_T_ values were used for the statistical analysis and in order to calculate mean values and SD or SEM, which were then converted into fold changes. The results were visualized using GraphPad Prism Version 9, showing mean ± SD for in vitro data and mean ± SEM for in vivo data. Statistical tests were calculated with GraphPad Prism Version 9 or R Version 4. An ANOVA with Dunnett’s post-hoc test was applied for the analyses of cytotoxicity and of the gene expression in triple cultures. In all other cases *t*-tests were performed. A *p*-value of <0.05 was considered statistically significant.

## 3. Results

### 3.1. ENM Characterization and Sedimentation

The hydrodynamic agglomerate diameters of CeO_2_ and SiO_2_ in E12 cell exposure medium were measured by DLS (Table 2). For TiO_2_ and Ag, these data were published previously [52]. For all four ENM the mean agglomerate diameters were in the range between 260 nm and 330 nm. 

**Figure 1:**
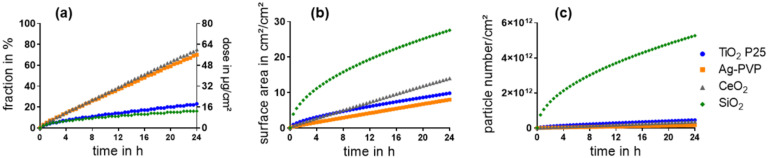
Sedimentation of ENM within 24 h was estimated with the ISDD model based on an initially administered dose of 80 µg/cm^2^. The sedimented fraction in % (**a left y-axis**), sedimented mass in µg/cm^2^ (**a right y-axis**), surface area of the sedimented particles (**b**), and sedimented particle number per cm^2^ (**c**) were calculated.

These results were used as inputs for the ISDD model to simulate the sedimentation of the ENM in E12 cell monoculture experiments (Figure 1a). The modelling results indicated two ENM with a relatively slow sedimentation, TiO_2_ and SiO_2_, whereas Ag and CeO_2_ both sedimented rather rapidly. Interestingly, SiO_2_ showed substantially higher values concerning the surface area and number of sedimented particles than the other ENM (Figure 1b,c). After 4 h the number of sedimented SiO_2_ particles was factor 15–66 and after 24 h factor 12–33 higher than of the other ENM.

### 3.2. Characterisation of the In Vitro Models

The mucus production of the E12 cell models as well as the constitutive expression of IL-8 and mucins was evaluated in all four in vitro models. Growth of E12 cells at confluence was accompanied by the increased production of acidic and neutral mucus. Confluence also came along with elevated constitutive expression levels of all investigated mucin genes. In stable triple cultures, all investigated mucin genes were lower in expression than in confluent E12 cells. The inflammatory state of triple cultures was associated with increased expression levels of IL-8 and particularly of MUC1.

#### 3.2.1. Mucus Production

**Figure 2:**
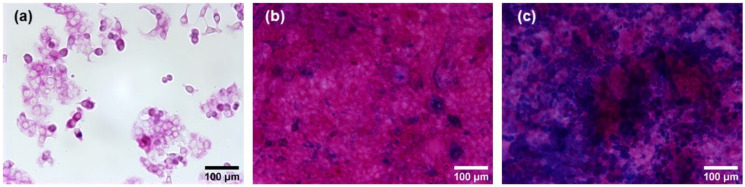
Alcian blue staining and PAS reaction on pre-confluent and confluent E12 cells. The cells were grown for two days (**a**), 11 days (**b**), and 22 days (**c**) post-seeding. Alcian blue was applied to assess acidic mucus. The PAS reaction yielded a pink color on neutral mucus. Images were acquired at 20× magnification.

The secretion and distribution of mucus in pre-confluent and confluent E12 cells was examined by Alcian blue staining and PAS reaction. Representative images of E12 cells grown for two days, 11 days, and 22 days are shown in Figure 2.

For pre-confluent E12 cells, no blue dye indicative of acidic mucus was present, while all cells exhibited a weak pink color (Figure 2a). For confluent E12 cells grown for 11 days post-seeding, blue spots of acidic mucus were observed (Figure 2b), which increased to a coverage of around 50% of the cell layer after 22 days (Figure 2c). On all confluent cells not covered by acidic mucus, a strong pink color was observed that pointed towards the presence of significant amounts of neutral mucus. Based on these results, it was inferred that the differentiation process of E12 cells triggered by confluence was not completed at 11 days post-seeding. Therefore, cells grown for 22 days post-seeding were used in the following experiments. The term ‘confluent E12 cells’ from here on refers to E12 cells grown 22 days post-seeding. 

#### 3.2.2. Gene Expression Profile

To further characterize the in vitro models, the constitutive gene expression of the five mucins and IL-8 was qualitatively assessed. The measured absolute C_T_-values of untreated cells were used as a rough estimate of the constitutive gene expression in pre-confluent and confluent E12 cells as well as in stable and inflamed triple cultures (Table S4). In each of the in vitro models, the constitutive expression of all investigated genes was above the limit of detection (C_T_-value 40); only in two of six experiments with stable triple cultures, MUC1 could not be detected. Apart from few exceptions, in all models the constitutive expression of IL-8, MUC1 and MUC2 was low and the expression of MUC5AC, MUC13 and MUC20 was high or very high. Remarkably, MUC2 was highly expressed in confluent E12 cells. In the inflamed triple culture, both IL-8 and MUC1 were expressed at a high level.

**Figure 3:**
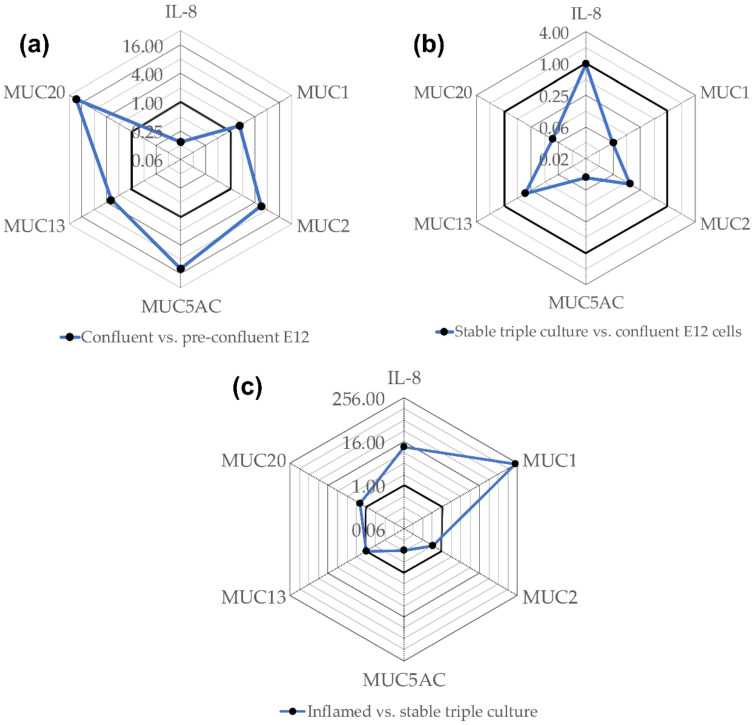
These radar charts show the gene expression in confluent E12 cells compared to pre-confluent E12 cells (**a**), in stable triple cultures compared to confluent E12 cells (**b**) and in inflamed compared to stable triple cultures (**c**) depicted as fold change. C_T_-values measured for untreated E12 cells and triple cultures were averaged. The fold change was calculated as ratio of the C_T_-value of one model and the C_T_-value of the next less complex model. The bold lines indicate fold change 1.0. Values closer to the center mean lower expression in the more complex model and vice versa.

The C_T_ values of the unexposed controls of each in vitro model were compared to those of the next complex model in order to roughly evaluate differences in the expression of mucins and IL-8 (Figure 3). The gene expression of all investigated mucins was upregulated in confluent E12 cells in a range of fold changes between 1.7 and 27.5 for MUC1 and MUC20, respectively (Figure 3a). The gene expression of IL-8, however, was downregulated. In comparison to the confluent E12 cells, the expression of all mucin genes was lower in the stable triple culture while there was no difference in IL-8 expression (Figure 3b). The most striking difference between the stable and inflamed triple culture was the more than hundred-fold higher expression of MUC1 in inflamed conditions (Figure 3c). MUC2 and MUC5AC were slightly higher and MUC13 and MUC20 slightly lower expressed in the inflamed triple culture. As anticipated, also the expression of IL-8 was remarkably increased under inflamed conditions. 

### 3.3. Effects of ENM on E12 Cells

#### 3.3.1. Cytotoxicity

**Figure 4:**
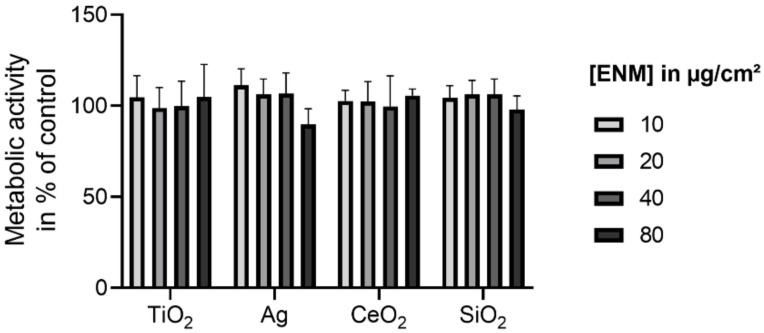
Relative viabilities of pre-confluent E12 cells upon exposure to TiO_2_, Ag, CeO_2_, and SiO_2_. Cells were exposed to concentrations in the range of 0–80 µg/cm^2^ for 24 h. The cell viability examined with the WST-1 assay was normalized to the negative control. The values are plotted as mean ± SD of *N* ≥ 3 independent experiments. An ANOVA with Dunnett’s post-hoc test was performed.

To select a non-cytotoxic ENM concentration for further investigation, the WST-1 assay was performed. The relative viabilities of pre-confluent E12 cells exposed to TiO_2_, Ag, CeO_2_, and SiO_2_ are presented in Figure 4. 

For all investigated ENM the viability was not significantly reduced up to the highest assessed concentration of 80 µg/cm^2^. Thus, for the following experiments 80 µg/cm^2^ was selected as the non-cytotoxic exposure concentration. This was also applied to confluent E12 cells as confluence was reported to be associated with lower susceptibility to cytotoxic effects [55,56,57].

#### 3.3.2. Gene Expression of IL-8 and Mucins in E12 Cell Monocultures

**Figure 5:**
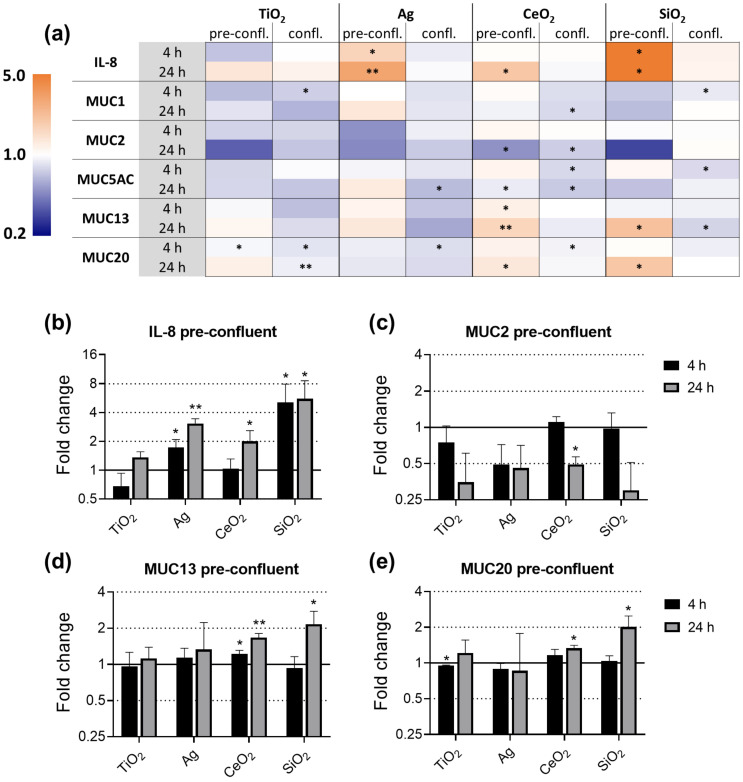
Gene expression of IL-8 and mucins in E12 cells upon exposure to TiO_2_, Ag, CeO_2_, and SiO_2_. Pre-confluent and confluent E12 cells were exposed to 80 µg/cm^2^ ENM for 4 h and 24 h. The relative gene expression of IL-8, MUC1, MUC2, MUC5AC, MUC13, and MUC20 was assessed by qRT-PCR. The results were normalized to the unexposed negative controls and β-Actin as reference gene. The depicted fold changes with SD were derived from the mean and SD of the ΔΔC_T_-values of *N* = 3 independent experiments (*t*-test; * *p* ≤ 0.05; ** *p* ≤ 0.01). The gene expression of all investigated genes is depicted as heatmap (**a**). As an extract, the gene expression of IL-8 (**b**), MUC2 (**c**), MUC13 (**d**) and MUC20 (**e**) in pre-confluent E12 cells is depicted separately.

Pre-confluent and confluent E12 cells were exposed to ENM for 4 h and 24 h and the gene expression was assessed by qRT-PCR (Figure 5). ENM effects were stronger and of a different nature, comparing pre-confluent to confluent E12 cells. While in pre-confluent E12 cells significant ENM induced upregulations occurred, we solely observed downregulations in confluent E12 that were relatively weak with a maximum foldchange of 0.65 in MUC5AC expression following Ag exposure. Trends that all ENM had in common as well as effects that were ENM specific were observed. The trends ENM had in common were the upregulation of IL-8 and the downregulation of MUC2 in pre-confluent E12 cells exposed for 24 h. The extent and ENM specificities of effects on MUC13 and MUC20 in pre-confluent E12 cells were highly similar to each other. In confluent E12 cells exposed for 24 h, MUC1 was specifically downregulated by CeO_2_. 

After 4 h of exposure, Ag and SiO_2_ upregulated IL-8. Although all ENM upregulated the expression of IL-8 in pre-confluent E12 cells after 24 h exposure, remarkable differences in the strength of the effects were observed between the ENM (Figure 5a,b). While SiO_2_ exposure induced a 5.6-fold upregulation, the impact of TiO_2_ was weak and not statistically significant. In strong contrast, none of the ENM had an effect on the IL-8 expression in confluent E12 cells. 

Significant effects on the expression of MUC1 were only seen in confluent E12 cells. At the 24 h timepoint, the MUC1 expression was specifically downregulated 1.3-fold by CeO_2_ exposure. 

As there was a trend to upregulate IL-8 in 24 h exposed pre-confluent cells, there also was a trend of all investigated ENM to downregulate MUC2 (Figure 5c). The effect only reached significance for CeO_2_. The trend was also observed in confluent E12 cells, though weaker and with the exception of SiO_2_.

The effects on the gene expressions of MUC13 and MUC20 in pre-confluent E12 cells were similar to each other (Figure 5d,e). The expression of both MUC13 and MUC20 was increased after exposure to CeO_2_ and to a higher extent after exposure to SiO_2_. With the exception of the CeO_2_ effect on MUC13, these effects were observed after exposure for 24 h but not after 4 h. In response to Ag, no effect was observed on the expression of both genes. TiO_2_ caused a marginal but significant downregulation of MUC13 after 4 h. In contrast to pre-confluent E12 cells, no congruent effects on the expression of MUC13 and MUC20 were detected in confluent E12 cells.

### 3.4. Effects of ENM on Stable and Inflamed Triple Cultures

With Ag and SiO_2_, we continued to test their effects on the more complex and more work-intensive stable and inflamed intestinal in vitro triple culture models.

**Figure 6:**
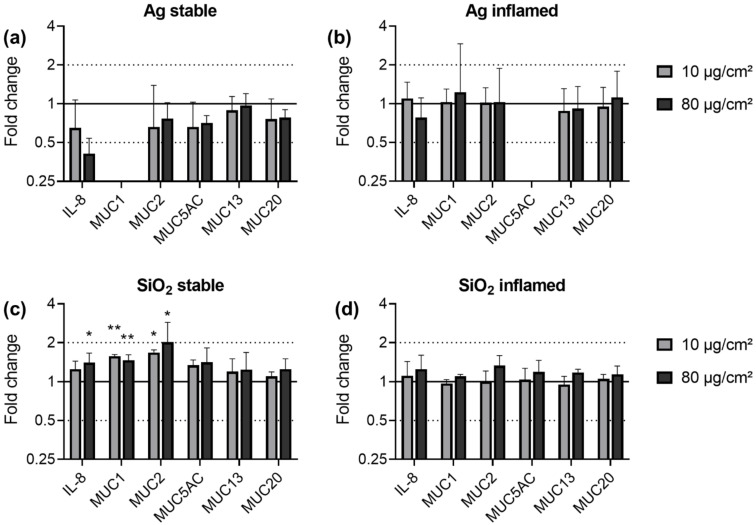
Gene expression of IL-8 and mucins in stable (**a**,**c**) and inflamed (**b**,**d**) triple cultures upon exposure to Ag (**a**,**b**) and SiO_2_ (**c**,**d**). Triple cultures were exposed to 10 and 80 µg/cm^2^ ENM for 24 h. The relative gene expression of IL-8, MUC1, MUC2, MUC5AC, MUC13, and MUC20 was assessed by qRT-PCR. The results were normalized to the unexposed negative controls and β-Actin as reference gene. The depicted fold changes with SD were derived from the mean and SD of the ΔΔC_T_-values of *N* = 3 independent experiments (*t*-test; * *p* ≤ 0.05; ** *p* ≤ 0.01).

The exposure of both triple culture models to Ag did not have an effect on any of the investigated genes (Figure 6a,b). Because of expression levels below the limit of detection, the MUC1 expression could not be assessed in stable cultures exposed to Ag. The MUC5AC data for the Ag exposed inflamed culture are absent as the DNA contamination analysis did not meet the reliability criteria for all three independent experiments. In stable triple cultures, SiO_2_ exposure caused upregulations of IL-8, MUC1 and MUC2 by a maximum of two-fold. No differences between low and high dose effects were observed; for the lower dose of 10 µg/cm^2^ SiO_2_, the increase in IL-8 did not reach statistical significance. SiO_2_ did not have an effect on the expression of any of the other investigated genes in the stable triple culture and on none of the analyzed genes in the inflamed triple culture. 

### 3.5. Gene Expression in the Ileum of Mice upon ENM Exposure

The constitutive gene expression of the mucin genes as well as of the murine IL-8 homologs macrophage inflammatory protein (Mip)-2 and keratinocyte-derived chemokine (Kc) expression was classified in vivo. Except for Muc5AC, the constitutive expression of all investigated genes was above the limit of detection (Table S5). Muc2 and Muc13 were highly expressed. The expression of all other investigated genes was low. Since Muc5AC could not be detected in the majority of measurements, effects of ENM on its expression were not assessed.

**Figure 7:**
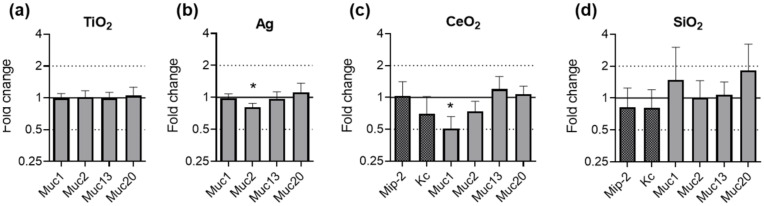
Gene expression of mucins in murine ileal tissues after ENM exposure. Female and male C57BL/6J mice were fed with feed pellets containing either no additive, 1.0% TiO_2_ (**a**) or 0.2% Ag (**b**) for 28 days. Female C57BL/6J mice were fed with feed pellets containing either no additive, 1.0% CeO_2_ (**c**) or 1.0% SiO_2_ (**d**) for 21 days. The relative gene expression of Muc1, Muc2, Muc13 and Muc20 was assessed by qRT-PCR. The results for the ENM exposed mice were normalized to those in the control mice using Rplp0 as reference gene. The depicted fold changes with SEM were derived from the mean and SEM of the ΔΔC_T_-values of *N* ≥ 5 independent experiments (*t*-test; * *p* ≤ 0.05).

Mice were exposed to TiO_2_ and Ag for 28 days and to CeO_2_ and SiO_2_ for 21 days. The effects on the gene expression in the ileal tissues are summarized in Figure 7. The effects of TiO_2_ and Ag on Mip-2 and Kc have been published earlier [52] and are shown in Figure S1.

No significant effects on the expression of any of the investigated genes was observed after exposure to TiO_2_ (Figure 7a). Upon exposure to Ag, the gene expression of Muc2 was significantly downregulated by fold change 0.81 (Figure 7b). The exposure of mice to CeO_2_ caused a significant two-fold downregulation of Muc1 (Figure 7c). SiO_2_ exposure did not lead to a change in the expression of any of the investigated genes (Figure 7d). 

## 4. Discussion

We assessed the impact of well-investigated model ENM on the gene expression of five mucins along with the chemokine IL-8 or its murine homologs in four in vitro models of different complexity and in mice. For pre-confluent and confluent E12 cells, the ENM concentration of 80 µg/cm^2^ was selected as no cytotoxicity was observed. In accordance with the dose in the E12 cell experiments and with our previous investigations, 10 and 80 µg/cm^2^ were used in the triple culture models [52]. As elucidated earlier [50], for the exposure of mice, ENM were incorporated into feed pellets to facilitate a realistic oral exposure scenario. The in vivo doses of TiO_2_, CeO_2_ and SiO_2_ of 1.0% were chosen to match the maximum permitted level of TiO_2_ in food defined by the United States Food and Drug Administration [58], whereas for Ag a lower dose of 0.2% was selected because of its higher oral toxicity [59,60,61]. It needs to be pointed out that we did not test food additives but four of the most-investigated model ENM in order to facilitate the comparability to other studies.

The individual mucins were investigated for different reasons: MUC2—the main secreted mucin—and MUC13—the main transmembrane mucins—are essential for the integrity of the intestinal mucus barrier; a distortion of their expression increases the susceptibility to develop intestinal inflammations [34,42,62,63]. The gastric mucins MUC1 and MUC5AC have been shown to differ in expression in case of an intestinal inflammation [34]. While MUC5AC is considered protective, alterations in the MUC1 expression in both directions have been associated with intestinal infections and inflammation [37,38,39,64,65,66]. The transmembrane mucin MUC20 was reported to be upregulated in the course of an intestinal infection or inflammation [35,36]. IL-8 was evaluated since it is a main messenger molecule of intestinal epithelial cells and is associated with the grade of inflammation in inflammatory bowel disease (IBD) [67,68].

In addition to 24 h ENM exposure, we also assessed the 4 h timepoint in order to cover potentially strong transient responses to ENM exposure in E12 cells. However, we merely found very weak transient effects of unknown biological relevance. Therefore, the discussion of the in vitro data is focused on the effects after 24 h of exposure. 

### 4.1. ENM and Model Dependent Effects on IL-8 Expression

While for all investigated ENM the same trends on the expression of IL-8 and MUC2 were observed in pre-confluent and confluent E12 cells, the extent of the effects differed between the models and particularly between the ENM. In pre-confluent E12 cells, Ag caused a stronger upregulation of IL-8 than TiO_2_. This is in concordance with our previous finding that Ag but not TiO_2_ increased the IL-8 secretion from pre-confluent E12 cells and pre-confluent Caco-2 cells [52]. The strikingly greater effects of SiO_2_ in the in vitro models might be explained by the manifold higher number of sedimented SiO_2_ particles and the associated higher surface area of sedimented particles compared to the other ENM [69,70].

In strong contrast to the pre-confluent E12 cells, none of the ENM affected the IL-8 expression in confluent E12 cells, which agrees with studies on Caco-2 cells: Susewind et al. exposed confluent Caco-2 cells to Ag [71]. For concentrations up to 625 μg/cm^2^ they reported an absence of effects on the expression and secretion of IL-8. Furthermore, Gerloff et al. reported an upregulation of the IL-8 expression in pre-confluent, but not in confluent Caco-2 cells exposed to 20 μg/cm^2^ SiO_2_ for 4 h [57]. 

Mirroring the SiO_2_ induced upregulation of IL-8 we observed in the stable triple culture, Ude et al. demonstrated the secretion of IL-8 from Caco-2/E12 cell co-culture exposed to 15.63 μg/cm^2^ SiO_2_ for 24 h [72]. In contrast, Ag exposure did not increase the IL-8 gene expression in both triple culture models, which aligns with previous findings on the absence of an Ag induced IL-8 secretion from these models [52]. 

### 4.2. ENM and Model Dependent Effects on MUC2 Expression

There was a trend of all investigated ENM to downregulate the MUC2 expression. Though weaker, this trend persisted in confluent E12 cells exposed to TiO_2_, Ag, and CeO_2_. On the contrary, in the stable triple culture and in mice, the effects on MUC2 were ENM specific. As for IL-8, SiO_2_ but not Ag upregulated MUC2 in the stable triple culture. In mice, only Ag significantly downregulated the expression of Muc2. While Jeong et al. observed decreased replenishment of mucus in ileal goblet cells that was in line with our results, Williams et al. stated that Ag did not influence the ileal Muc2 expression in rats [73,74]. 

Previously we demonstrated an increased abundance of the short chain fatty acid (SCFA) producing microbial genus *Roseburia* in the Ag exposed female mice [50]. Accordingly, increased intestinal SCFA levels could have contributed to the currently observed downregulation of Muc2. Indeed, changing concentrations of propionate and butyrate have been demonstrated to influence Muc2 expression [75,76,77,78]. However, Seo et al. associated treatment with *Roseburia* to a rescuing effect on the expression levels of Muc2 following exposure of mice and gut organoids to ethanol [79]. This also highlights the inconclusive implications of the microbiome and its metabolites for intestinal toxicity, rendering it a factor worth assessing and controlling in vitro, e.g., by cultivating cells in the presence of microbial metabolites such as SCFAs [17]. 

Especially in the context of the above discussed effects on IL-8 and the dependence of the mucus barrier integrity on MUC2, the ENM induced downregulations of MUC2 imply the hazard of an inflammation [80,81]. In addition, Muc2 deficiency has been connected to an aberrant inflammatory response including increased Il-8 release and to changes of metabolic pathways with an associated formation of preneoplastic lesions [82,83]. Muc2 deficient mice are more prone to ileal infections and develop spontaneous ileal tumors [40,41]. Hence, an ENM induced Muc2 downregulation might increase the susceptibility for such adverse outcomes. However, the constitutive Muc2 expression in vivo was high and the extent of the observed downregulations did not bring the expression levels close to a deficiency. Moreover, a downregulation of Muc2 gene expression does not necessarily go along with decreased protein secretion levels [84].

### 4.3. ENM Specific and Potentially Hazardous Effects on MUC1 and MUC13

Any change in the gene expression of MUC1 must be examined carefully as its balance seems to be essential for cell homeostasis: An overexpression of MUC1 has been linked to the development of colorectal cancer and an acceleration of intestinal inflammatory processes [65,66], whereas Muc1 deficiency was connected to an increased susceptibility to the invasion of the intestinal epithelium by pathogens [37,38,39]. Therefore, the significant downregulation of MUC1 in confluent E12 cells and in mice following CeO_2_ exposure in combination with the low constitutive level of Muc1 expression may give cause for concern. The same applies to the upregulation of MUC1 in the SiO_2_ exposed stable triple culture, especially as it is paralleled by the upregulation of IL-8 and MUC2: Increased mucus levels have the potential to amplify the secretion of IL-8, to promote the recruitment of neutrophils and, therefore, the development of inflammatory processes [85]. 

In pre-confluent E12 cells, exposure to CeO_2_ and SiO_2_ caused upregulation of MUC13. According to Sheng et al., an upregulation of MUC13 was described in Ulcerative Colitis patients though no causative relation was reported [34]. Moreover, they stated that the secretion of the murine IL-8 homolog Mip-2α by Tnfα was lower in the intestine of Muc13 deficient mice [63]. However, they also described that Muc13 deficiency in mice aggravates dextran sulfate sodium-induced colitis, attributing a protective function to Muc13 [42]. Considering this potential protective function of Muc13, it is not possible to estimate if an upregulation would lead to an adverse outcome. 

For SiO_2_ and CeO_2_ exposed pre-confluent E12 cells, the upregulation of MUC13 coincided with the upregulation of MUC20. Like MUC13, MUC20 is upregulated in the intestinal tissue of IBD patients [34,36]. In addition, Cairns et al. reported the concomitant upregulation of MUC13 and MUC20 upon infection of E12 cells with *Helicobacter pylori*, indicating a potential mechanistic connection between these two genes [35]. 

Multiple mechanisms have been connected to the regulation of the expression of mucins, out of which ENM induced oxidative stress may be particularly relevant for the present effects [86,87,88,89]. Furthermore, the NFκB signaling pathway that has been shown to influence MUC1 expression and also plays and important role for the expression of IL-8 may be involved [39]. Whether or not cellular uptake of ENM uptake is of importance for these mechanisms and for the resulting effects on the mucin expression should be investigated in future projects, especially because changes in the mucus profile can in turn influence the ENM uptake [90].

Altogether, the moderate ENM effects in the advanced models do not indicate strong toxicity. Yet, further research is required to clarify whether the alterations of MUC1, MUC2 and MUC13 expression are physiologically relevant for the susceptibility towards intestinal infections and inflammations. Moreover, future studies will need to address the effects of prolonged ENM exposure.

### 4.4. Model Comparisons

A particular advantage of our study design has been that the same batches of ENM were tested in vitro and in a physiological exposure situation in mice, which enables more direct comparisons. In addition to pre-confluent and confluent E12 cells in monoculture, we evaluated ENM effects on triple cultures of Caco-2, E12 and differentiated THP-1 cells, both in stable and inflamed states. Co-cultures including Caco-2 and HT29-MTX(-E12) cells are frequently used as in vitro models of the mucus-covered intestinal epithelium for toxicity testing [17,27,52,72,91]. Yet, little research is available on the response of HT29-MTX(-E12) cells to ENM exposure.

#### 4.4.1. Comparison of In Vitro Models

Comparing pre-confluent to confluent E12 cells, strong differences in ENM effects were seen. No upregulation was found in confluent E12 cells. The downregulatory effects we observed were relatively weak. The strongest effects in pre-confluent E12 cells were induced by SiO_2_ exposure, which were all absent in confluent E12 cells. As mentioned above, for Caco-2 cells decreasing susceptibilities towards ENM effects have been associated with confluence [57,92]. A possible reason for the higher resistance of confluent cells in general may be stronger cell-cell contacts [93]. Concerning HT29-MTX(-E12) cells, an explanation may be the increasing levels of mucus production and secretion accompanying confluence which have also been reported by others [45,94]. Notably, Reuter et al. were able to confirm the protective function of confluent E12 cells’ mucus layer towards the bacterial toxin colibactin [95].

Confluent E12 cells revealed higher expression levels of each investigated mucin compared to the stable triple culture model. This underlines the role of E12 cells as goblet-cell model whereas the triple culture model mimics an epithelial layer consisting mainly of enterocytes. In contrast to the downregulations in confluent E12 cells, we only found upregulations in the stable triple culture model. Whether this difference is related to the presence of Caco-2 cells (~90%), which may react unlike E12 cells, or a changed behavior of E12 cells in the presence of Caco-2 cells remains unknown.

The most outstanding difference between the inflamed and the stable triple culture model was the strongly increased expression of MUC1. Moreover, we observed moderate decreases in the expression of MUC2 and MUC5AC under inflamed conditions. These findings agree with studies on IBD patients and mice with an artificially induced colitis [34,96,97,98]. Contrasting our results, IBD has formerly been associated with higher expression levels of MUC5AC [34,98]. 

Without an inflamed mouse model, the inflamed triple culture cannot be directly compared to the in vivo data. Still, the absence of effects in the inflamed triple culture suggests no increased susceptibility towards the here tested ENM in the course of inflammatory processes. 

#### 4.4.2. Suitability of the In Vitro Models in Comparison to the In Vivo Data

In ENM fed mice, we observed two significant effects on the expression of the investigated genes: the downregulation of Muc1 upon CeO_2_ exposure and the downregulation of Muc2 upon Ag exposure. The specific effect of CeO_2_ on MUC1 could also be found in confluent E12 cells, and thus supports the relevance of this model for in vitro nanosafety research. In addition, having a high constitutive expression of MUC2 and MUC13 of the investigated in vitro models confluent E12 cells best represented the constitutive gene expression in vivo. 

Nevertheless, it makes sense to not consider the goblet-like E12 cells in isolation but rather in combination with Caco-2 cells as a model for enterocytes. As presented and discussed earlier by Kämpfer et al. [52], the mucus production and secretion patterns of the stable triple culture closely resembled that of the small intestinal epithelium of a healthy mouse. 

Overall, effects were relatively strong in pre-confluent E12 cells and almost absent in the more advanced models. The pre-confluent cells might overestimate ENM effects compared to the in vivo situation. These observations correspond to our former findings that Ag caused pro-inflammatory effects in pre-confluent Caco-2 and E12 cells while the stable triple culture model was predictive of the absence of those effects in vivo [52]. 

## 5. Conclusions

While the absence of effects in the inflamed triple culture indicates that an acute inflammation does not increase the susceptibility to ENM for the investigated endpoints, the exposure to ENM may pose the hazard of increasing the susceptibility to intestinal infections and the development of intestinal inflammation. We conclude this from upregulations of IL-8 and MUC13, as well as alterations of the MUC1 and MUC2 expression. In this context, CeO_2_ and Ag demand particular attention, especially considering their effects on MUC1 and MUC2 in vivo. Furthermore, we conclude that growth at confluence is crucial to prevent an overestimation and misinterpretation of ENM effects from in vitro models. Accordingly, confluent E12 cells and stable triple cultures reflected the in vivo effects well. Moreover, the capacity of the triple culture model to resemble changes in the mucin profile of patients and mice with an inflamed intestine emphasizes its suitability to investigate the toxicity of ENM in both healthy and inflamed phenotypes.

## Figures and Tables

**Table 1:** Cell number, well volume and well filling height for different plate formats according to the respective size of the well surface.

Plate Format	Well Surface	Cells per Well	Volume	Filling Height
6-Well	9.6 cm^2^	30 × 10^4^	3.0 mL	3.2 mm
96-Well	0.32 cm^2^	1 × 10^4^	0.1 mL	3.2 mm

**Table 2:** Agglomerate diameter of CeO_2_ and SiO_2_ determined via DLS measurement.

ENM	Hydrodynamic Diameter [nm]	PDI
CeO_2_	284 ± 13	0.264
SiO_2_	264 ± 9	0.392

## Data Availability

The data presented in this study are openly available in Mendeley Data at doi:10.17632/znd6v266cx.1.

## Supplementary Materials

The following are available online at https://www.mdpi.com/article/10.3390/nano11102621/s1, Table S1: Characteristics of pristine ENM as described by Bredeck et al. [50], Table S2: Primer sequences for human genes, Table S3: Primer sequences for murine genes, Table S4: Estimated constitutive gene expression in pre-confluent and confluent E12 cells as well as in stable and inflamed triple cultures, Table S5: Estimated constitutive gene expression in murine ileal tissue, Figure S1: Estimated constitutive gene expression in murine ileal tissue.
