# Supplementary material for: Ingested Engineered Nanomaterials Affect the Expression of Mucin Genes—An In Vitro-In Vivo Comparison
Source: Nanomaterials (Basel). 2021 Oct 6;11(10):2621. doi: 10.3390/nano11102621 (PMC8537393; doi:10.3390/nano11102621)
Supplement: Supplementary file 1 [file nanomaterials-11-02621-s001.zip › nanomaterials-1383619-supplementary.pdf]

Supplementary Materials

Table S1. Characteristics of pristine ENM as described by Bredeck et al. [50].

ENM [1]	Name, Source	Mean Diameter (nm)	Mode Diameter (nm)	Sigma (fit) [2]
TiO_2	NM105, JRC	26.2 ± 10.7	21.0	1.39
Ag	#576832-5G, Sigma-Aldrich	40.2 ± 17.6	32.0	1.40
CeO_2	NM212, JRC	35.4 ± 17.0	28.7	1.38
SiO_2	#S5130, Sigma-Aldrich	12.9 ± 4.9	11.0	1.32

[1] Engineered nanomaterials (ENM) used in the respective studies were analyzed by scanning electron microscopy as described by Bredeck et al. [50]. [2] Standard deviation of logarithmic values.

Table S2. Primer sequences for human genes.

Gene		Sequence (5′ → 3′)	Working Conc. (nM)	Amplicon Length (bp)
β-Actin	fw	CCTGGCACCCAGCACAAT	60	70
	rv	GCCGATCCACACGGAGTACT	60	
IL-8	fw	ACTCCAAACCTTTCCACCC	60	168
	rv	CCCTCTTCAAAAACTTCTCCAC	60	
MUC1	fw	AGACGTCAGCGTGAGTGATG	37.5	139
	rv	GACAGCCAAGGCAATGAGAT	37.5	
MUC2	fw	GTCCGTCTCCAACATCACCT	60	287
	rv	GCTGGCTGGTTTTCTCCTCT	60	
MUC5AC	fw	CAGCACAACCCCTGTTTCAAA	60	100
	rv	GCGCACAGAGGATGACAGT	37.5	
MUC13	fw	CAGAGACAGCCAGATGCAAA	60	126
	rv	CGGAGGCCAGATCTTTACTG	37.5	
MUC20	fw	GTGCAGGTGAAAATGGAGGT	60	299
	rv	ACGCAGTAAGGAGACCTGGA	37.5	

Table S3. Primer sequences for murine genes.

Gene		Sequence (5′ → 3′)	Working Conc. (nM)	Amplicon Length (bp)
Rplp0	fw	CCACGAAAATCTCCAGAGGC	100	164
	rv	AATGCTGCCGTTGTCAAACA	375	
Muc1	fw	GCTGGACATCTTTCCAACCC	100	137
	rv	TACTGCCATTACCTGCCGAA	100	
Muc2	fw	CCATTGAGTTTGGGAACATGC	100	104
	rv	TTCGGCTCGGTGTTCAGAG	375	
Muc5AC	fw	AAAGACACCAGTAGTCACTCAGCAA	375	139
	rv	CTGGGAAGTCAGTGTCAAACCA	100	
Muc13	fw	TCGTCATGTGTGAAAGGAACG	100	228
	rv	CTTTGTAGCATCACGCATTGC	100	
Muc20	fw	GCACCAGCAAAAACCCAAAC	60	153
	rv	TCAGCCGTACAAGGAGGAAG	60	

Table S4. Estimated constitutive gene expression in pre-confluent and confluent E12 cells as well as in stable and inflamed triple cultures.

	IL-8	MUC1	MUC2	MUC5AC	MUC13	MUC20
Pre-confluent E12	+	-	-	++	+	+
Confluent E12	-	-	+	++	+	++
Triple culture stable	-	-	-	++	+	+
Triple culture inflamed	+	+	-	+	+	+

According to the mean of the measured C_T-values of non-exposed cells, the gene expression was classified into the following categories: very high gene expression at C_T-values 19–25 (++); high gene expression at C_T-values 25–31 (+); low gene expression at C_T-values 31–37 (-); very low gene expression at C_T-values >37 (--).

Table S5. Estimated constitutive gene expression in murine ileal tissue.

Mip-2	Kc	Muc1	Muc2	Muc5AC	Muc13	Muc20
-	-	-	+	--	+	-

According to the mean of the measured C_T-values of non-exposed mice, the gene expression was classified into the following categories: very high gene expression at C_T-values 19–25 (++); high gene expression at C_T-values 25–31 (+); low gene expression at C_T-values 31–37 (-); very low gene expression at C_T-values >37 (--).

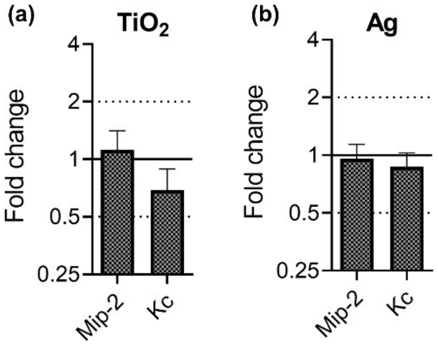

Figure S1. Gene expression of Mip-2 and Kc in murine ileal issues after ENM exposure as published previously [52]. Female and male C57BL/6J mice were fed with feed pellets containing either no additive, 1.0% TiO_2 (**a**) or 0.2% Ag (**b**) for 28 days. The relative gene expression was assessed by qRT-PCR. The results were normalized to the unexposed negative controls and Rplp0 as reference gene. The depicted fold changes with SEM were derived from the mean and SEM of the $\Delta\Delta C_T$-values of N ≥ 5 independent experiments (*t*-test; * $p \leq 0.05$).
